# The Role of Hyperthermic Intrathoracic Chemotherapy (HITHOC) in Thoracic Tumors

**DOI:** 10.3390/cancers16142513

**Published:** 2024-07-11

**Authors:** Federica Danuzzo, Maria Chiara Sibilia, Sara Vaquer, Andrea Cara, Enrico Mario Cassina, Lidia Libretti, Emanuele Pirondini, Federico Raveglia, Antonio Tuoro, Francesco Petrella

**Affiliations:** Department of Thoracic Surgery, Fondazione IRCCS San Gerardo dei Tintori, 20900 Monza, Italy; federica.danuzzo@unimi.it (F.D.); maria.sibilia@unimi.it (M.C.S.); sara.vaquer@unimi.it (S.V.); andrea.cara@irccs-sangerardo.it (A.C.); enricomario.cassina@irccs-sangerardo.it (E.M.C.); lidia.libretti@irccs-sangerardo.it (L.L.); emanuele.pirondini@irccs-sangerardo.it (E.P.); federico.raveglia@irccs-sangerardo.it (F.R.); antonio.tuoro@irccs-sangerardo.it (A.T.)

**Keywords:** mesothelioma, pleural malignancies, hyperthermic intrathoracic chemotherapy (HITHOC)

## Abstract

**Simple Summary:**

Hyperthermic intrathoracic chemotherapy (HITHOC) is an intraoperative and topical administration of chemotherapeutic drugs with simultaneous warming of the thoracic cavity. This procedure was first described by Spratt in 1980 as a thermal transfusion infiltration system, performed on canine models to treat malignant effusions of metastatic abdominal cancers. The main tumors causing malignant pleural effusion, which have been seen to benefit from hyperthermic intrathoracic chemotherapy, are mesothelioma, thymic malignancies and lung cancer: despite the high prevalence of MPE in patients with metastatic breast and ovarian cancers, there are still inadequate data on the use of HITHOC as a treatment option for these malignancies.

**Abstract:**

Pleural mesothelioma (PM) is a rare but aggressive thoracic tumor with a poor prognosis. Multimodal treatment—including induction chemotherapy, aggressive surgical resection, radiotherapy and immunotherapy in selected cases—currently represents the best therapeutic option. Single-center studies advocate hyperthermic intrathoracic chemotherapy (HITHOC) during surgical resection as an additional therapeutic option, although its impact on post-operative morbidity and survival has not yet been evaluated on a larger scale. HITHOC can be applied not only in the case of mesothelioma, but also in the case of thymoma with pleural involvement or—in very selected cases—in patients with secondary pleural metastases. Despite favorable outcomes and reduced clinical risks, there is no uniform approach to HITHOC, and a wide variety of indications and technical applications are still reported. Based on available data, HITHOC seems to offer a clear benefit in regard to overall survival of all mesothelioma patients; however, multicenter randomized controlled trials are required to validate and standardize this approach. The aim of this review is to focus on the present role of HITHOC in thoracic tumors with pleural involvement as well as on future challenges, particularly in the light of possible combined therapy of thoracic tumors still presenting poor prognoses.

## 1. Introduction

Pleural malignancies and thoracic neoplasms involving pleura currently represent a hard challenge for clinicians and surgeons for reasons of disease aggressiveness, rapid inauspicious evolution, poor prognoses and severe general clinical conditions. Hyperthermic intrathoracic chemotherapy (HITHOC), which was first introduced at the end of the 1990s, is an intraoperative administration of chemotherapeutic drugs with simultaneous warming of the thoracic cavity. The main application of HITHOC worldwide is the treatment of malignant pleural mesothelioma, which often occurs with pleural effusion: several studies demonstrated the synergetic effect of HITHOC and surgical resection in terms of less post-operative morbidity and better survival. Thymic malignancies and lung cancer have also been proven to benefit from HITHOC as adjuvant treatment, but only in strictly selected patients: there is still a lack of data on the use of HITHOC as a treatment option for patients affected by metastatic breast and ovarian cancer with pleural involvement.

This review aims to provide an overview on the present role of HITHOC in the field of multimodality treatments of thoracic tumors with pleural involvement, focusing on principal features, techniques, current applications and future perspectives and challenges.

## 2. Malignant Pleural Effusions: Therapeutic Approach and Framework

Malignant pleural effusion (MPE) is the accumulation of fluid containing neoplastic cells in the thoracic cavity: it is a quite common manifestation and complication of advanced tumors, associated with high morbidity and mortality rates of 37% at 30 days and 77% at 1 year [1].

The development of MPE is an expression of advanced-stage carcinoma with poor prognosis, severely affecting the clinical status and the quality of life of patients.

An MPE develops as a result of increased production of the pleural fluid and decreased re-absorption, which may be due to direct neoplastic infiltration and consequent absorption by the lymphatic system removal or to local inflammation and increased capillary permeability resulting from neoplastic involvement of the pleural surface [2]. It has been demonstrated that neoplastic patients—presenting with pleural effusions—show high serum and plasma levels of vascular endothelial growth factor (VEGF), which is the most important angiogenic factor produced by neoplastic cells, promoting vascular permeability, endothelial cell migration and tumor metastatic progression [3].

Pleural effusion can be the first manifestation of pleural mesothelioma (PM) or can be secondary to other malignancies: lung and breast cancer are the most common tumors that metastasize to the pleura in men and women, respectively, following by Hodgkin/non-Hodgkin lymphoma representing the most common cause of pleural effusion in young adults [4]. Fifteen percent of patients with non-small-cell lung cancer (NSCLC) present MPE at diagnosis, and 50% of these patients will eventually develop malignant pleural effusion [5,6].

MPE is a widespread clinical condition, affecting more than 150,000 people in the USA and 50,000 in the UK each year: it is estimated that about 15% of people affected by advanced cancer will develop pleural effusion because of pleural tumor infiltration [2,7,8].

Patients with MPE usually complain of dyspnea, cough and chest pain, and it could also be associated with weight loss and asthenia: the severity of symptoms correlates with the amount of pleural effusion.

The primary goal in the approach of MPE is establishing a diagnosis and improving clinical status and the quality of life by relieving symptoms, especially respiratory ones: thoracentesis and placement of thoracic drainages are diagnostic and palliative maneuvers and allow cytological analysis of the fluid for diagnosis to improve dyspnea and cough, to monitor the re-accumulation of the effusion and to assess for non-expandable lungs. Chemical pleurodesis is a palliative option, consisting in the delivery of talc into the thoracic cavity to allow fusion of parietal and visceral pleura, thus preventing fluid re-accumulation: it can be administered as talc slurry (suspension made from talc and saline solution) through a chest tube or as talc poudrage during thoracoscopy. Parietal and visceral pleural apposition is indispensable to obtain pleurodesis, so in the case of incomplete lung re-expansion, the latest evidence-based guideline from the American Thoracic Society in 2018 recommended the positioning of an indwelling pleural catheter (IPC), a tunneled drainage that can be positioned in out-patients designed to stay in place for longer periods [9].

During the past year, several perioperative or intraoperative intra-cavitary therapies have been proposed to improve the loco-regional effect of surgery. The rationale is to administer cytotoxic agents in the pleural cavity and to reach the microscopically remaining tumor cells, where a direct and more efficient effect can be achieved, limiting the systemic adverse effects. In addition to chemotherapy, antiseptic povidone, photodynamic therapy and immunotherapies have been used [10,11,12,13]. However, although several studies described and tested the efficacy of intrapleural injection of chemotherapy agents as adjuvant treatment in the management of MPE in terms of greater pathological response and improvement in survival, the results are still based on low-quality retrospective data, and intracavitary treatments are recommended to be used within a clinical trial.

The potential and promising benefits of this technique are to enhance the local cytotoxic effect after debulking surgery with less systemic toxicity and improving clinical status, quality of life and time of disease-free survival. 

## 3. Historic Background and First Experiments: What Do We Currently Know?

**Figure 1:**
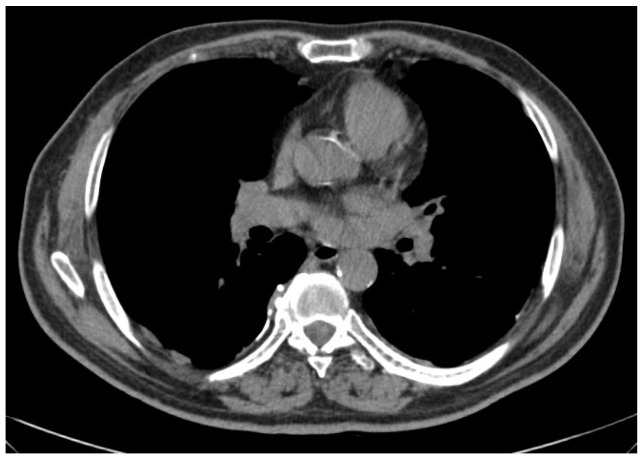
Limited epithelioid mesothelioma (right side) amenable of pleurectomy/decortication.

**Figure 2:**
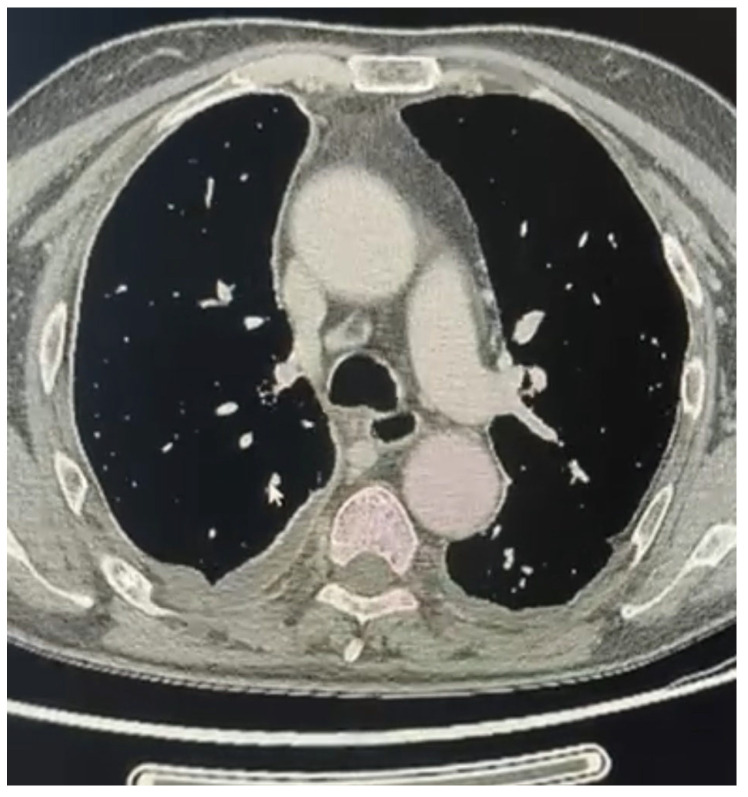
Extended sarcomathoid mesothelioma with lymph nodes involvement (right side) not amenable of pleurectomy/decortication.

Hyperthermic intrathoracic chemotherapy (HITHOC) is an intraoperative and topical administration of chemotherapeutic drugs with simultaneous warming of the thoracic cavity. The concept of hyperthermic intra-cavity chemotherapy was initially proposed in the peritoneum (hyperthermic intraperitoneal chemotherapy, HIPEC) to treat metastatic abdominal tumors as a coadjuvant treatment of extensive surgical resection with cytoreductive intent, with the purpose of improving survival. This procedure was first described by Spratt in 1980 as a thermal transfusion infiltration system, performed on canine models to treat malignant effusions of metastatic abdominal cancers [14]. Since then, HIPEC has been increasingly used and has achieved an important role in the multimodal management and treatment of primary and secondary peritoneal tumors. The technique consists in administering chemotherapy agents into the peritoneal cavity using the principle of the peritoneal-plasma barrier, which limits the absorption of drugs from the peritoneal cavity into the blood, improving the local action and reducing systemic toxicity [15]. The elevated temperature of fluid administration (41 to 43 °C)—which has a synergistic effect with several chemotherapy agents enhancing their absorption—creates vascular stasis of vascular tumor microcirculation, resulting in greater destruction of neoplastic cells [16,17,18]. Intrathoracic chemotherapy was first described in 1955 by Bateman and colleagues, who reported local intra-cavitary injection of triethylene thiophosphoramide into the pleural, abdominal or pericardial cavity after removal of fluid effusion in ambulatory patients. They found better results in malignant pleural effusion as compared to patients presenting ascites, probably because of anatomical features of the thoracic and abdominal cavities and different neoplastic nodules and fluid distribution. Bateman et al. concluded that local injection of chemotherapy not only permitted the administration of higher doses of drug than via the systemic route, but also produced a better local pathological response and effect on distant disease. Moreover, they did not find any early clinical side effects (which allows this procedure to be performed on out-patients) or any changes in tumor-free tissue following phosphoramide intracavity administration [19]. In 2008, Matsuzaki et al. first treated a group of six patients affected by malignant pleural mesothelioma at advanced stages with cisplatin-based intrapleural perfusion hyperthermo-chemotherapy, at 43 degrees for 120 min. They collected tumor cells from pleural effusions pre- and postperfusion and measured the apoptotic index, detecting increased apoptosis after perfusion with a peak after 24 h [20]. Subsequent in vitro experiments demonstrated the antineoplastic effect of only hyperthermia on mesothelioma cells for temperatures higher than 45 °C: instead, the combination of hyperthermia with cytostatic agents (mostly cisplatin) increased the cytotoxic effect also at lower temperatures (42 °C) [21]. Furthermore, Larisch and colleagues proved the considerable efficacy of HITHOC in terms of local toxicity, performing ex vivo and in vitro experiments to evaluate both concentration and penetration of chemotherapy agents in lung tissue after decortication, incubation with cisplatin and hyperthermic exposure (at 37, 42 or 45 °C for 60 min) [22]. The maximum penetration depth of cisplatin was found to be 7.5 mm, while a previous ex vivo study estimated the penetration depth of cisplatin in non-decorticated lung tissue to be 3–4 mm at 42 °C [21]. They also evaluated survival rates of PM cells at different depths, comparing decorticated tissue samples and non-decorticated ones, finding that more tumor cells were killed at greater depths after decortication [22] (Figure 1 and Figure 2).

## 4. Features and Techniques

It has been demonstrated that the combination of hyperthermia and intrapleural cisplatin (200 mg/m^2^ of body surface area (BSA), 120 min, 43 °C) induces significant apoptosis of neoplastic cells [22]. The apoptosis curve begins to rise immediately after perfusion and peaks 24 h after the intrathoracic administration. The mechanisms by which antineoplastic agents work in this context to induce apoptosis are mitochondrial depolarization, phosphatidylserine translocation and caspase activation. On the other hand, hyperthermia determines protein denaturation of the cancer cells, inhibits RNA synthesis, modifies the DNA synthesis, promotes mitosis arrest, increases reactive oxygen species (ROS) production and the number of unstable lysosomes; it increases membrane permeability and improves membrane transport, modifying cell metabolism, the excretion and the pharmacokinetics of drugs, thereby increasing the cytotoxicity of chemotherapeutic agents [23]. It should also be noted that cancer cells are more thermosensitive, and thus undergo apoptosis at temperatures between 41 and 43 °C (unlike healthy cells): several treatments administrated at temperatures ranging from 40 to 44 degrees have been proven to be cytotoxic for cells in a microenvironment with low pH and low oxygen partial pressure, which are typical of tumor tissue, for the reason of poor blood perfusion [24,25]. Considering all these aspects, the synergistic action that chemotherapy and hyperthermia play in determining the increased local cytotoxicity of neoplastic cells becomes clear. Furthermore, the combination of two cytotoxic drugs also seems to further enhance the effectiveness of the procedure: the most frequently used agent is cisplatin, which can be used in combination with doxorubicin or mitomycin C with hyperthermia at temperature ranges between 41 and 43 °C, with standard time for infusion of 60–90 min across several studies. In vitro experiments demonstrated that washing with cisplatin at a concentration of 0.05 mg/mL for 60 min at 42 °C ensures a tissue concentration of 2.4 µg/mL with a penetration depth of 3–4 mm to 7.5 mm in decorticated lung tissue [21,22]. These data are encouraging and could prove the potential of HITHOC as an adjuvant treatment of pleural malignancies in combination with debulking surgery, especially for thymic advanced tumors and mesothelioma, whose most widespread treatment is pleurectomy decortication. Surgical treatment in these patients fails to be radical because of the characteristics and stage of disease, and therefore has the sole objective of minimizing the disease burden: this is why hyperthermic intrathoracic chemotherapy can enhance the surgical action of increasing local cytotoxicity by acting on the residual superficial neoplastic cells. Even if higher doses of chemotherapy agents need to be applied, it has been observed that the systemic absorption of the drug remains very low, which makes HITHOC a relatively safe and particularly suitable treatment for patients with multiple comorbidities who would be especially affected by the systemic toxicity of cisplatin: in serum samples, the concentration of cisplatin, which is administered in the pleural cavity, reached only a fraction of about 2% with a peak occurring 60 min after the procedure [21].

## 5. Benefits and Side Effects

In a 2017 systematic review and meta-analysis considering 21 articles, Zhao et al. reported no perioperative or HITHOC-associated (30-day or 90-day) mortality [26]. Nevertheless, hyperthermic intrathoracic chemotherapy does have potential common side effects due to both chemotherapy agents and hyperthermic application. Patients can complain of pain and discomfort in the treated area, particularly in the days following the procedure, associated with fatigue sometimes lasting several weeks; nausea and vomiting as well as anemia, leukopenia, thrombocytopenia, increased risk of infections and bleeding; kidney and liver toxicity leading to potential dysfunction or damage are induced by chemotherapy and seem to be dose-dependent risks; hyperthermia can lead to changes in electrolyte levels, which need to be carefully monitored and corrected; respiratory complications, dyspnea, pulmonary edema or pleural effusion may occur as a direct consequence of local treatment.

Ashraf-Kashani et al. reported significant hemodynamic instability induced by hyperthermic agents resulting in vasodilatation, cardiac warming and compression of mediastinal vessels evolving sometimes into cardiac asystole during the HITHOC procedure [27]. Similarly, Kerscher and colleagues reported unexpected side effects including high pressure of the intrathoracic and central venous system and the potential risk of systemic hyperthermia during cytoreduction surgery and HITHOC and a rate of 10% of an impairment of coagulation in post-operative laboratory analysis [28].

Currently, pleurectomy-decortication or extra-pleural pneumonectomy are the major strategies of treatment for malignant pleural mesothelioma: other therapies, such as radiotherapy and systemic chemotherapy, are combined with surgery as perioperative adjuncts in order to enhance the therapeutic effect of surgery, as well as to control the relapse of disease. A 2017 systematic review and meta-analysis demonstrated that patients affected by mesothelioma can benefit from HITHOC treatment either intra-operatively or post-operatively. They analyzed five studies, which compared the therapeutic results of patients who received hyperthermic intrapleural chemotherapy during cytoreductive surgery or extra-pleural pneumonectomy to that of patients who did not receive HITHOC: both overall survival and disease-free survival were longer in the patients treated additionally with hyperthermic intrapleural chemotherapy [13,26,29,30,31,32].

Other subsequent studies consistently show the role and efficacy of HITHOC in the setting of multimodality treatment of malignant pleural mesothelioma, demonstrating significantly improved overall survival and disease-free survival combining cytoreductive surgery with hyperthermic intrapleural chemotherapy; this suggests relevant benefits of HITHOC compared to surgery alone: the median overall survival was found to range from 11 to 75 months and disease-free survival from 7.2 to 57 months, appearing to be superior to surgical treatment alone (overall survival 5–36 months and disease-free interval 12.1–21 months). Moreover, a higher dose of hyperthermic intrathoracic chemotherapy was associated with an improvement in overall survival compared with a lower dose (18–31 months versus 6–18 months) [33,34]. A more recent meta-analysis by Järvinen and colleagues could not definitively conclude that all mesothelioma patients benefitted from HITHOC treatment, although they found a statistically significant effect in favor of HITHOC, which is particularly notable in patients with the epithelioid subtype of PM, which responds better to the combined treatment [35] (Table 1).

Some studies have shown that HITHOC is also used in the multimodal treatment of advanced-stage thymic tumors, which are frequently and belatedly misdiagnosed and often confused with pleural dissemination, especially when not associated with myasthenia gravis; moreover, even locally advanced thymomas are characterized by a high rate of pleural or pericardial relapses. Surgery has always been considered the gold standard of thymoma treatment, even in the case of local intrathoracic dissemination or relapses in the context of multidisciplinary protocols with chemotherapy and/or radiotherapy. In the early 1990s, the introduction of HITHOC as a coadjuvant to surgery changed the treatment prospects of these patients with stage IVa thymomas or thymoma pleural recurrences, giving hope in terms of less toxicity than systemic chemotherapy and providing better local disease control in patients [46]. In 2001, Refaely et al. investigated the role of HITHOC in 15 patients affected by stage IVa thymoma or thymic carcinoma treated by surgery with positive resection margins (R1/R2) followed by HITHOC (cisplatin at a dosage between 100 and 200 mg/m^2^ at about 43 °C for 1 h). They did not describe any complications or platinum-related toxicity and reported survival rates of 90% at 3 years from surgery and 70% at 5 years [47]. Ried et al. published their experience on patients with stage III and IVa thymomas who underwent post-operative HITHOC: 89% of the patients were alive without disease with a median survival of 25 months [39]. In 2017, Maury et al. published a study on 19 patients operated for thymoma pleural recurrences receiving HITHOC protocol with cisplatin and mitomycin perfused for 60 min at about 42 °C. They reported seven patients (37%) with tumor recurrence with a median disease-free survival of 53 months, while the median local disease-free interval was 41 months; median overall survival was 63 months and 1- and 5-year survival rates were 93% and 86%, respectively [48]. The largest publication group on this topic was made by Aprile and colleagues in 2020: it is a retrospective study of a 12-year experience comparing outcomes of surgery + HITHOC and surgery alone for the treatment of thymoma pleural recurrences. The HITHOC protocol consisted of cisplatin and epirubicin at 42 °C for 60 min: they found similar post-operative morbidity and no procedure-related mortality, similar survival rates, but significantly different local disease-free intervals [49] (Table 2).

Lastly, some authors have also started to propose the application of cytoreductive surgery followed by HITHOC to improve local pleural control and overall survival for selected patients affected by advanced NSCLC. Shigemura et al. first published a pilot study with five patients on HITHOC and pleuro-pneumonectomy for patients with advanced lung cancer and carcinomatous pleuritis (cN0-1, M0-1). They first performed surgery for pleural biopsies and thoracoscopic intrapleural perfusion hyperthermic chemotherapy (TIPHC): subsequently, they performed cytological examination of pleural effusion to confirm the negative and after 2 weeks from TIPHC, pleuro-pneumonectomy was scheduled. The mean survival time observed was 19 months, and the longest was 32 months [55]. In 2019, Migliore and colleagues conducted a systematic review to evaluate the efficacy of debulking surgery and HITHOC as a treatment for selected patients with NSCLC and malignant pleural effusion [56,57]. They selected four articles with a total of 21 patients who had undergone debulking surgery and HITHOC: surgery ranged from wedge resection to pneumonectomy and pleurectomy with HITHOC was performed in all patients for 1 h; the median survival was 18 months (range 1–74 months) with 62% of patients alive at 1 year and 28.5% alive at 2 years. Due to insufficient data, they were not able to perform a meta-analysis and concluded that despite some encouraging and stimulating evidence, the effectiveness of the procedure for selected patients with advanced lung cancer was still weak, needing some randomized controlled trials to clearly evaluate the benefit of a new therapeutic approach (Table 3).

## 6. Conclusions

From the first experiments to more recent data, numerous studies on the use of intrathoracic chemotherapy have been carried out, but most of them are case series, feasibility studies or retrospective studies with small numbers of patients. The main tumors causing malignant pleural effusion, which have been seen to benefit from hyperthermic intrathoracic chemotherapy, are mesothelioma, thymic malignancies and lung cancer: despite the high prevalence of MPE in patients with metastatic breast and ovarian cancers, there are still inadequate data on the use of HITHOC as a treatment option for these malignancies [61] (Table 4). In the last few decades, hyperthermic intrathoracic chemotherapy has been reported to be a feasible and safe procedure and represents a promising therapeutic option in the context of a multimodality approach to thoracic-spread malignancies involving the pleura. Since patients affected by pleural malignancy have a poor prognosis with reduced expectation of life and serious general clinical conditions, all the therapeutic instruments available are palliative. HITHOC allows better loco-regional tumor control, enhancing surgery treatment and improving disease-free and overall survival of patients with pleural malignancies. HITHOC has been proven to be a feasible and effective local procedure in the context of multimodal treatment strategies for PM, offering significant advantages in terms of survival and disease control compared to standard treatments alone; on the other hand, its role has still not been discussed in the latest guidelines of the task force of the ERS/EACTS/ESTS/ESCRO on treatment of PM [23]. The feasibility, efficacy and safety of this procedure have already been demonstrated with both ex vivo and in vivo studies, but there is still considerable divergence and heterogeneity of thought and clinical practice among clinicians approaching pleural malignancies. Currently, there is a lack of consensus and no guidelines regarding clinical protocols and techniques: there are still great differences in surgical aspects and indications, patient selection, the choice of antitumoral drugs perfused and their dosage, temperature, time of the perfusion and type of perfusion machine. The challenge of the coming years will be to design and conduct prospective multicenter randomized studies on large series of patients with pleural malignancies, in order to optimize and standardize the treatment protocols of HITHOC and expand its clinical application.

## Figures and Tables

**Table 1:** Studies on HITHOC application for MPM [33].

Author	Year	Study Type	Patients	Surgery + HITHOC	Follow-Up, Survival Rates and Oncological Outcomes
Monneuse [36]	2003	Prospective	17	PP, P/D, Wedge	Mean follow-up time: 89 months Median OS: 18 months
Richards [37]	2006	Prospective	44	EPD	Median OS: 13 months Median DFI: 7.2 months
van Sandick [29]	2008	Retrospective	20	EPP, P/D	Mean follow-up time: 89 months Median OS: 11 months Median DFI: 8 months
Tilleman [31]	2009	Prospective	92	EPP	Mean follow-up time: 31 months Median OS: 13.1 months Median DFI: 15.3 months
Zellos [38]	2009	Prospective	29	EPP	Median OS: 20 months Median DFI: 16 months
Ried [39]	2013	Prospective	8	EPD/PD	Mean follow-up time: 17 months Median OS: 18 months Median DFI: 4.5 months
Sugarbaker [30]	2013	Retrospective	72	EPP/PD	Mean follow-up time: 54 months Median OS: 35.3 months Median DFI: 27.1 months
Ishibashi [40]	2015	Prospective	4	P/D	Median OS: 17.2 months Median DFI: 12.5 months
Migliore [41]	2015	Prospective	6	P/D, Wedge, EPD	Median OS: 21.5 months
Işik [32]	2016	Retrospective	36	EPP, P/D	Median OS: 27 months
Ambrogi [42]	2018	Retrospective	49	P/D, Wedge	Mean follow-up time: 69 months Median OS: 22 months
Luzzi [43]	2018	Prospective	41	EPD	Median OS: 25 months
Burt [44]	2018	Prospective	104	EPP, P/D	Mean follow-up time: 51 months Median OS: 20.3 months Median DFI: 10.7 months
Patel [45]	2019	Retrospective	5	EPP, EPD	Mean follow-up time: 9 months Median OS: 15 months Median DFI: 24 months
Klotz [34]	2019	Retrospective	71	P/D	Median OS: 16.1 months

DFI, disease-free interval; OS, overall survival; EPP, extrapleural pneumonectomy; EPD, extended pleurectomy decortication; PD, pleurectomy-decortication; PP, partial pleurectomy.

**Table 2:** Studies on HITHOC application for thymoma IV stage and pleural recurrences [46].

Author (Year)	Patients	Disease	Surgery + HITHOC	Follow-Up, Survival Rates and Oncological Outcomes	HITHOC-Related Side Effects (%)
Yellin (2001) [50]	7	Thymoma IV stage	EPP, P/D, Wedge	Mean follow-up: 60.2 months 5 alive without disease 74.3%	1 (14.3)
Refaely (2001) [47]	10	Thymoma IV stage	EPP, P/D	3-year OS: 90% 5-year OS: 70%	3 (30.0)
De Bree (2002) [51]	3	Pleural recurrences	EPP, P/D	Mean follow-up: 18 months OS: 100%, contralateral recurrence at 13 months 33.3%	1 (33.3)
Ried (2013) [52]	8	Thymoma IV stage	P/D	Mean follow-up: 22 months OS: 87.5% DFS: 87.5% (1 relapse after 13 months)	0
Yellin (2013) [53]	31	Pleural recurrences + Thymoma IV stage	PP, P/D, Wedge	Mean follow-up: 62 months 5-year OS 67%, 10-year OS 56% 5-year DFS = 48%, 10-year DFS = 18%	-
Yu (2013) [54]	4	Thymoma IV stage + Pleural recurrences	Cytoreduction surgery	Follow-up: 1–4 years DFS = 100%	2 (50.0)
Ried (2014) [39]	9	Thymoma IV stage	P/D	Mean follow-up: 29.3 months Median OS: 25 months DFS: 89%, Recurrence rate: 22.7%	0
Maury (2017) [48]	19	Pleural recurrences	PP, P/D, Wedge	Median follow-up: 39 months Median OS: 63 months 1-year OS: 93%, 5-year OS: 86% Median DFS: 53 months	3 (15.9)
Aprile (2020) [49]	27	Pleural recurrences	PP, P/D	Mean follow-up: 70.9 months Mean OS: 153.1 months, 10-year OS: 77% 2 recurrence rate: 44.4%	0

DFS, disease-free survival; OS, overall survival; EPP, extrapleural pneumonectomy; PD, pleurectomy-decortication; PP, partial pleurectomy.

**Table 3:** Studies on HITHOC application for MPE from local advanced lung cancer.

Author	Year	Study Type	Patients	Treatment	Disease	Median Survival Time
Shigemura [55]	2003	Prospective	5	HITHOC + Surgery	Local advanced lung cancer	19 months (32 months the longest)
Monneuse [36]	2003	Retrospective	3	HITHOC + Surgery	Local advanced lung cancer	33 months
Kimura [58]	2010	Prospective	5	HITHOC + Surgery	Local advanced lung cancer	25 months
Işık [32]	2013	Retrospective	19	HITHOC + Surgery	Local advanced lung cancer+ Secondary neoplasm	15 months
Moon [59]	2015	Retrospective	10	HITHOC	Local advanced lung cancer + breast cancer + ovarian cancer	3–7-month recurrence-free rates: 86.9 and 73.9%
Migliore [56]	2015	Retrospective	2	HITHOC + Surgery	Local advanced lung cancer	21 months
Yi [60]	2016	Retrospective	33	HITHOC + Surgery	Local advanced lung cancer	Overall survival at 3 years 38.6%

**Table 4:** Studies on HITHOC application for MPE from secondary neoplastic tumors.

Author	Year	Study Type	Patients	Disease	Local Control Rate Assessed
Fracchia [62]	1970	Retrospective	138	Breast cancer	Yes
Markman [63]	1984	Retrospective	4	Breast/ovarian cancer	Yes
Contegiacom [64]	1987	Retrospective	21	Breast cancer	Yes
Rusch [65]	1991	Prospective	46	Breast/ovarian cancer	-
Kan [66]	1993	Retrospective	67	Breast cancer	-
Aasebo [67]	1997	Prospective	30	Breast/ovarian cancer	Yes
Shoji [68]	2002	Prospective	22	Breast cancer	-
Mitamura [69]	2009	Case report	1	Ovarian cancer	Yes
Jones [70]	2010	Prospective	15	Breast/ovarian cancer	-
Singh [71]	2014	Retrospective	4	Ovarian cancer	Yes
Feng [72]	2017	Retrospective	80	Breast cancer	Yes
Jun [73]	2017	Case report	1	Ovarian cancer	-
Zhang [74]	2021	Randomized controlled trial	84	Breast cancer	-

The efficacy of the HITHOC was assessed and reported in seven studies (169 patients with breast cancer and 8 with ovarian cancer). The local control rates of malignant pleural mesothelioma were 59.1% for breast cancer patients and 87.5% for ovarian cancer at 4 weeks after treatment [61].

## Data Availability

Data available on request.

## References

[1] DeBiasi E.M., Pisani M.A., Murphy T.E., Araujo K., Kookoolis A., Argento A.C., Puchalski J. (2015). Mortality among patients with pleural effusion undergoing thoracentesis. Eur. Respir. J..

[2] Feller-Kopman D., Light R. (2018). Pleural Disease. N. Engl. J. Med..

[3] Chen Y.A.O., Mathy N.W., Lu H. (2018). The role of VEGF in the diagnosis and treatment of malignant pleural effusion in patients with non-small cell lung cancer (Review). Mol. Med. Rep..

[4] Lat T., Paul M. (2024). Malignant Effusion (Archived). StatPearls [Internet].

[5] Masago K., Fujimoto D., Fujita S., Hata A., Kaji R., Ohtsuka K., Okuda C., Takeshita J., Katakami N. (2015). Response to bevacizumab combination chemotherapy of malignant pleural effusions associated with non-squamous non-small–cell lung cancer. Mol. Clin. Oncol..

[6] Clive A.O., Kahan B.C., Hooper C.E., Bhatnagar R., Morley A.J., Zahan-Evans N., Bintcliffe O.J., Boshuizen R.C., Fysh E.T.H., Tobin C.L. (2014). Predicting survival in malignant pleural effusion: Development and validation of the LENT prognostic score. Thorax.

[7] Clive A.O., Jones H.E., Bhatnagar R., Preston N.J., Maskell N. (2020). Interventions for the management of malignant pleural effusions: A network meta-analysis. Cochrane Database Syst. Rev..

[8] Rahman N.M., Ali N.J., Brown G., Chapman S.J., Davies R.J.O., Downer N.J., Gleeson F.V., Howes T.Q., Treasure T., Singh S. (2010). Local anaesthetic thoracoscopy: British Thoracic Society Pleural Disease Guideline 2010. Thorax.

[9] Feller-Kopman D.J., Reddy C.B., DeCamp M.M., Diekemper R.L., Gould M.K., Henry T., Iyer N.P., Lee Y.C.G., Lewis S.Z., Maskell N.A. (2018). Management of malignant pleural effusions. An official ATS/STS/STR clinical practice guideline. Am. J. Respir. Crit. Care Med..

[10] Bertoglio P., Aprile V., Ambrogi M.C., Mussi A., Lucchi M. (2018). The role of intracavitary therapies in the treatment of malignant pleural mesothelioma. J. Thorac. Dis..

[11] Friedberg J.S., Simone C., Culligan M.J., Barsky A.R., Doucette A., McNulty S., Hahn S.M., Alley E., Sterman D., Glatstein E. (2017). Extended Pleurectomy-Decortication-Based Treatment for Advanced Stage Epithelial Mesothelioma Yielding a Median Survival of Nearly Three Years. Ann. Thorac. Surg..

[12] Lucchi M., Chella A., Melfi F., Dini P., Ambrogi M., Fino L., Fontanini G., Mussi A. (2007). A phase II study of intrapleural immuno-chemotherapy, pleurectomy/decortication, radiotherapy, systemic chemotherapy and long-term sub-cutaneous IL-2 in stage II-III malignant pleural mesothelioma. Eur. J. Cardio-Thorac. Surg..

[13] Lang-Lazdunski L., Bille A., Lal R., Cane P., McLean E., Landau D., Steele J., Spicer J. (2012). Pleurectomy/decortication is superior to extrapleural pneumonectomy in the multimodality management of patients with malignant pleural mesothelioma. J. Thorac. Oncol..

[14] Spratt J.S., Adcock R.A., Sherrill W., Travathen S. (1980). Hyperthermic peritoneal perfusion system in canines. Cancer Res..

[15] Jacquet P., Sugarbaker P.H. (1996). Peritoneal-plasma barrier. Cancer Treat. Res..

[16] de Bree E., Helm C.W. (2012). Hyperthermic intraperitoneal chemotherapy in ovarian cancer: Rationale and clinical data. Expert. Rev. Anticancer Ther..

[17] de Bree E., Tsiftsis D.D. (2007). Principles of perioperative intraperitoneal chemotherapy for peritoneal carcinomatosis. Advances in Peritoneal Surface Oncology.

[18] Brown S.L., Hunt J.W., Hill R.P. (1992). Differential thermal sensitivity of tumour and normal tissue microvascular response during hyperthermia. Int. J. Hyperth..

[19] Bateman J.C., Moulton B., Larsen N.J. (1955). Control of neoplastic effusion by phosphoramide chemotherapy. AMA Arch. Intern. Med..

[20] Matsuzaki Y., Tomita M., Shimizu T., Hara M., Ayabe T., Onitsuka T. (2008). Induction of apoptosis by intrapleural perfusion hyperthermo-chemotherapy for malignant pleural mesothelioma. Ann. Thorac. Cardiovasc. Surg..

[21] Ried M., Lehle K., Neu R., Diez C., Bednarski P., Sziklavari Z., Hofmann H.S. (2015). Assessment of cisplatin concentration and depth of penetration in human lung tissue after hyperthermic exposure. Eur. J. Cardiothorac. Surg..

[22] Larisch C., Markowiak T., Loch E., Großer C., Bednarski P.J., Mueller K., Hofmann H.S., Ried M. (2021). Assessment of concentration and penetration depth of cisplatin in human lung tissue after decortication and hyperthermic exposure. Ann. Transl. Med..

[23] Migliore M., Ried M., Molins L., Lucchi M., Ambrogi M., Molnar T.F., Hofmann H.-S. (2021). Hyperthermic intrathoracic chemotherapy (HITHOC) should be included in the guidelines for malignant pleural mesothelioma. Ann. Transl. Med..

[24] Yi G.Y., Kim M.J., Kim H.I., Park J., Baek S.H. (2022). Hyperthermia Treatment as a Promising Anti-Cancer Strategy: Therapeutic Targets, Perspective Mechanisms and Synergistic Combinations in Experimental Approaches. Antioxidants.

[25] Ahmed K., Zaidi S.F. (2013). Treating cancer with heat: Hyperthermia as promising strategy to enhance apoptosis. J. Pak. Med. Assoc..

[26] Zhao Z.Y., Zhao S.S., Ren M., Liu Z.L., Li Z., Yang L. (2017). Effect of hyperthermic intrathoracic chemotherapy on the malignant pleural mesothelioma: A systematic review and meta-analysis. Oncotarget.

[27] Ashraf-Kashani N., Bell J. (2017). Haemodynamic changes during hyperthermic intra-thoracic chemotherapy for pseudomyxoma peritonei. Int. J. Hyperth..

[28] Kerscher C., Ried M., Hofmann H.S., Graf B.M., Zausig Y.A. (2014). Anaesthetic management of cytoreductive surgery followed by hyperthermic intrathoracic chemotherapy perfusion. J. Cardiothorac. Surg..

[29] van Sandick J.W., Kappers I., Baas P., Haas R.L., Klomp H.M. (2008). Surgical treatment in the management of malignant pleural mesothelioma: A single institution’s experience. Ann. Surg. Oncol..

[30] Sugarbaker D.J., Gill R.R., Yeap B.Y., Wolf A.S., DaSilva M.C., Baldini E.H., Bueno R., Richards W.G. (2013). Hyperthermic intraoperative pleural cisplatin chemotherapy extends interval to recurrence and survival among low-risk patients with malignant pleural mesothelioma undergoing surgical macroscopic complete resection. J. Thorac. Cardiovasc. Surg..

[31] Tilleman T.R., Richards W.G., Zellos L., Johnson B.E., Jaklitsch M.T., Mueller J., Yeap B.Y., Mujoomdar A.A., Ducko C.T., Bueno R. (2009). Extrapleural pneumonectomy followed by intracavitary intraoperative hyperthermic cisplatin with pharmacologic cytoprotection for treatment of malignant pleural mesothelioma: A phase II prospective study. J. Thorac. Cardiovasc. Surg..

[32] Isik A.F., Sanli M., Yilmaz M., Meteroglu F., Dikensoy O., Sevinc A., Camci C., Tuncozgur B., Elbeyli L. (2013). Intrapleural hyperthermic perfusion chemotherapy in subjects with metastatic pleural malignancies. Respir. Med..

[33] Dawson A.G., Kutywayo K., Mohammed S.B., Fennell D.A., Nakas A. (2023). Cytoreductive surgery with hyperthermic intrathoracic chemotherapy for malignant pleural mesothelioma: A systematic review. Thorax.

[34] Klotz L.V., Zimmermann J., Müller K., Kovács J., Hassan M., Koller M., Schmid S., Huppertz G., Markowiak T., Passlick B. (2024). Multimodal Treatment of Pleural Mesothelioma with Cytoreductive Surgery and Hyperthermic Intrathoracic Chemotherapy: Impact of Additive Chemotherapy. Cancers.

[35] Järvinen T., Paajanen J., Ilonen I., Räsänen J. (2021). Hyperthermic Intrathoracic Chemoperfusion for Malignant Pleural Mesothelioma: Systematic Review and Meta-Analysis. Cancers.

[36] Monneuse O., Beaujard A.C., Guibert B., Gilly F.N., Mulsant P., Carry P.Y., Benoit M., Glehen O. (2003). Long-Term results of intrathoracic chemohyperthermia (ITCH) for the treatment of pleural malignancies. Br. J. Cancer.

[37] Richards W.G., Zellos L., Bueno R., Jaklitsch M.T., Jänne P.A., Chirieac L.R., Yeap B.Y., Dekkers R.J., Hartigan P.M., Capalbo L. (2006). Phase I to II study of pleurectomy/decortication and intraoperative intracavitary hyperthermic cisplatin lavage for mesothelioma. J. Clin. Oncol..

[38] Zellos L., Richards W.G., Capalbo L., Jaklitsch M.T., Chirieac L.R., Johnson B.E., Bueno R., Sugarbaker D.J. (2009). A phase I study of extrapleural pneumonectomy and intracavitary intraoperative hyperthermic cisplatin with amifostine cytoprotection for malignant pleural mesothelioma. J. Thorac. Cardiovasc. Surg..

[39] Ried M., Potzger T., Sziklavari Z., Diez C., Neu R., Schalke B., Hofmann H.S. (2014). Extended surgical resections of advanced thymoma Masaoka stages III and IVa facilitate outcome. Thorac. Cardiovasc. Surg..

[40] Ishibashi H., Kobayashi M., Takasaki C., Okubo K. (2015). Interim results of pleurectomy/decortication and intraoperative intrapleural hyperthermic cisplatin perfusion for patients with malignant pleural mesothelioma intolerable to extrapleural pneumonectomy. Gen. Thorac. Cardiovasc. Surg..

[41] Migliore M., Calvo D., Criscione A., Palmucci S., Fuccio Sanzà G., Caltabiano R., Spatola C., Privitera G., Aiello M.M., Parra H.S. (2015). Pleurectomy/decortication and hyperthermic intrapleural chemotherapy for malignant pleural mesothelioma: Initial experience. Future Oncol..

[42] Ambrogi M.C., Bertoglio P., Aprile V., Chella A., Korasidis S., Fontanini G., Fanucchi O., Lucchi M., Mussi A. (2018). Diaphragm and lung-preserving surgery with hyperthermic chemotherapy for malignant pleural mesothelioma: A 10-year experience. J. Thorac. Cardiovasc. Surg..

[43] Luzzi L., Franchi F., Dapoto A., Ghisalberti M., Corzani R., Marrelli D., Marchetti L., Paladini P., Scolletta S. (2018). Hyperthermic intrathoracic chemotherapy after extended pleurectomy and decortication for malignant pleura mesothelioma: An observational study on outcome and microcirculatory changes. J. Thorac. Dis..

[44] Burt B.M., Richards W.G., Lee H.S., Bartel S., Dasilva M.C., Gill R.R., Jaklitsch M.T., Johnson B.E., Swanson S.J., Bueno R. (2018). A phase I trial of surgical resection and intraoperative hyperthermic cisplatin and gemcitabine for pleural mesothelioma. J. Thorac. Oncol..

[45] Patel M.D., Damodaran D., Rangole A., Shaikh S., Shah K., Bagwade R., Bhatt A. (2019). Hyperthermic intrathoracic chemotherapy (HITHOC) for pleural Malignancies-Experience from Indian centers. Indian J. Surg. Oncol..

[46] Aprile V., Bacchin D., Korasidis S., Ricciardi R., Petrini I., Ambrogi M.C., Lucchi M. (2021). Hyperthermic Intrathoracic Chemotherapy (HITHOC) for thymoma: A narrative review on indications and results. Ann. Transl. Med..

[47] Refaely Y., Simansky D.A., Paley M., Gottfried M., Yellin A. (2001). Resection and perfusion thermochemotherapy: A new approach for the treatment of thymic malignancies with pleural spread. Ann. Thorac. Surg..

[48] Maury J.-M., Drevet G., Tronc F., Girard N. (2017). Intra-Thoracic Chemo-Hyperthermia for pleural recurrence of thymomas. J. Thorac. Dis..

[49] Aprile V., Bacchin D., Korasidis S., Nesti A., Marrama E., Ricciardi R., Petrini I., Ambrogi M.C., Paladini P., Lucchi M. (2020). Surgical treatment of pleural recurrence of thymoma: Is hyperthermic intrathoracic chemotherapy worthwhile?. Interact. Cardiovasc. Thorac. Surg..

[50] Yellin A., Simansky D.A., Paley M., Refaely Y. (2001). Hyperthermic pleural perfusion with cisplatin: Early clinical experience. Cancer.

[51] de Bree E., van Ruth S., Baas P., Emiel J., van Zandwijk N., Witkamp A.J., Zoetmulder F.A. (2002). Cytoreductive surgery and intraoperative hyperthermic intrathoracic chemotherapy in patients with malignant pleural mesothelioma or pleural metastases of thymoma. Chest.

[52] Ried M., Potzger T., Braune N., Neu R., Zausig Y., Schalke B., Diez C., Hofmann H.S. (2013). Cytoreductive surgery and hyperthermic intrathoracic chemotherapy perfusion for malignant pleural tumours: Perioperative management and clinical experience. Eur. J. Cardiothorac. Surg..

[53] Yellin A., Simansky D.A., Ben-Avi R., Perelman M., Zeitlin N., Refaely Y., Ben-Nun A. (2013). Resection and heated pleural chemoperfusion in patients with thymic epithelial malignant disease and pleural spread: A single-institution experience. J. Thorac. Cardiovasc. Surg..

[54] Yu L., Jing Y., Ma S., Li F., Zhang Y.F. (2013). Cytoreductive surgery combined with hyperthermic intrapleural chemotherapy to treat thymoma or thymic carcinoma with pleural dissemination. OncoTargets Ther..

[55] Shigemura N., Akashi A., Ohta M., Matsuda H. (2003). Combined surgery of intrapleural perfusion hyperthermic chemotherapy and panpleuropneumonectomy for lung cancer with advanced pleural spread: A pilot study. Interact. Cardiovasc. Thorac. Surg..

[56] Migliore M. (2017). Debulking surgery and hyperthermic intrathoracic chemotherapy (HITHOC) for lung cancer. Chin. J. Cancer Res..

[57] Migliore M., Nardini M. (2019). Does cytoreduction surgery and hyperthermic intrathoracic chemotherapy prolong survival in patients with N0-N1 nonsmall cell lung cancer and malignant pleural effusion?. Eur. Respir. Rev..

[58] Kimura M., Tojo T., Naito H., Nagata Y., Kawai N., Taniguchi S. (2010). Effects of a simple intraoperative intrathoracic hyperthermotherapy for lung cancer with malignant pleural effusion or dissemination. Interact. CardioVascular Thorac. Surg..

[59] Moon Y., Kim K.S., Park J.K. (2015). Simple intrapleural hyperthermia at thoracoscopic exploration to treat malignant pleural effusion. J. Cardiothorac. Surg..

[60] Yi E., Kim D., Cho S., Kim K., Jheon S. (2016). Clinical outcomes of cytoreductive surgery combined with intrapleural perfusion of hyperthermic chemotherapy in advanced lung adenocarcinoma with pleural dissemination. J. Thorac. Dis..

[61] Karampinis I., Dionysopoulou A., Galata C., Almstedt K., Grilli M., Hasenburg A., Roessner E.D. (2022). Hyperthermic intrathoracic chemotherapy for the treatment of malignant pleural effusion caused by breast and ovarian cancer: A systematic literature review and pooled analysis. Thorac. Cancer..

[62] Fracchia A.A., Knapper W.H., Carey J.T., Farrow J.H. (1970). Intrapleural chemotherapy for effusion from metastatic breast carcinoma. Cancer.

[63] Markman M., Howell S.B., Green M.R. (1984). Combination intracavitary chemotherapy for malignant pleural disease. Cancer Drug Deliv..

[64] Contegiacomo A., Fiorillo L., De Placido S., Pagliarulo C., Iaffaioli R.V., Genua G., Giampaglia F., Palmieri G., Bianco A.R. (1987). The treatment of metastatic pleural effusion in breast cancer: Report of 25 cases. Tumori J..

[65] Rusch V.W., Figlin R., Godwin D., Piantadosi S. (1991). Intrapleural cisplatin and cytarabine in the management of malignant pleural effusions: A lung cancer study group trial. J. Clin. Oncol..

[66] Kan N., Kodama H., Hori T., Takenaka A., Yasumura T., Kato H., Ogawa H., Mukaihara S., Kudo T., Ohsumi K. (1993). Intrapleural adaptive immunotherapy for breast cancer patients with cytologically-confirmed malignant pleural effusions: An analysis of 67 patients in Kyoto and Shiga prefecture, Japan. Breast Cancer Res. Treat..

[67] Aasebø U., Norum J., Sager G., Slørdal L. (1997). Intrapleurally instilled mitoxantrone in metastatic pleural effusions: A phase II study. J. Chemother..

[68] Shoji T., Tanaka F., Yanagihara K., Inui K., Wada H. (2002). Phase II study of repeated intrapleural chemotherapy using implantable access system for management of malignant pleural effusion. Chest.

[69] Mitamura T., Hosaka M., Takeda M., Watari H., Sakuragi N. (2009). Intrathoracic injection of paclitaxel for a patient with stage IV serous ovarian cancer: A case report. Cancer Chemother. Pharmacol..

[70] Jones D.R., Taylor M.D., Petroni G.R., Shu J., Burks S.G., Daniel T.M., Gillenwater H.H. (2010). Phase I trial of intrapleural docetaxel administered through an implantable catheter in subjects with a malignant pleural effusion. J. Thorac. Oncol..

[71] Singh S., Armstrong A., Robke J., Waggoner S., Debernardo R. (2014). Hyperthermic intra-thoracic chemotherapy (HITeC) for the management of recurrent ovarian cancer involving the pleural cavity. Gynecol. Oncol. Case Rep..

[72] Feng X., Zhu L., Xiong X., Jiang H., Wu Z., Meng W., Xu Y., Zhang S., Ma S. (2018). Therapeutical effect of intrapleural perfusion with hyperthermic chemotherapy on malignant pleural effusion under video-assisted thoracoscopic surgery. Int. J. Hyperth..

[73] Jun S.Y., Seok Y.K., Kato T., Lee Y.H., Chong G.O., Lee Y.S., Cho Y.L., Hong D.G. (2017). Hyperthermic intrathoracic chemotherapy with cisplatin for ovarian cancer with pleural metastasis. Obstet. Gynecol. Sci..

[74] Zhang H., Jiang M., Gao L., Lin Z. (2021). The clinical efficacy of external application of mirabilite and rhubarb combined with intrathoracic chemotherapy in treating malignant pleural effusion: A prospective, randomized, controlled clinical trial. Medicine.

